# Community-based program to increase use of hearing conservation practices among farm and rural youth: a cluster randomized trial of effectiveness

**DOI:** 10.1186/s12889-020-08972-3

**Published:** 2020-06-03

**Authors:** Marjorie C. McCullagh, James J. Yang, Michael A. Cohen

**Affiliations:** School of Nursing, University of Michigan, 400 North Ingalls Street, Ann Arbor, MI 48109-5482 USA

**Keywords:** Noise exposure, Hearing loss, Farm youth, Children

## Abstract

**Background:**

Noise exposure and associated hearing loss affects an estimated 2 million farm youth who are exposed as farm residents, farm family workers, hired workers, children of migrant or seasonal workers, and farm visitors. Risk factors for farm youth include frequent exposure to high farm noise; farm work from an early age, and exposure to high recreational noise (e.g., firearms, ATVs, and personal listening devices).

**Methods:**

This study compared the effectiveness of two interventions and control. The programs included a community-based interactive youth educational program alone (Group A), a community-based interactive youth educational program followed by an Internet-based booster (Group B), and a no-interaction control (Group C). The study used a cluster randomized control design, with equal allocation ratio to each cluster, without blinding. Inclusion criteria included enrollment in grade 4, parental consent, English speaking, and attending a community-based educational event included in the cluster sampling. A total of 1979 youth were enrolled at 36 sites distributed across the 3 study arms in the following distribution: *N* = 662 in 13 sites (Group A), *N* = 680 in 12 sites (Group B), and *N* = 637 in 11 sites (Group C).

**Results:**

Comparison with pre-intervention data showed no difference in intent to use hearing conservation strategies in experimental groups. However, knowledge and attitudes toward hearing conservation were improved in the groups receiving the Internet-based booster. Participants reported frequent exposure to sources of hazardous noise (e.g., loud sporting events, firecrackers, personal listening devices).

**Conclusions:**

It is feasible and acceptable to incorporate hearing health education into an already existing system designed to deliver health and safety educational programming to farm and rural youth. The program was adopted by the partner agency for dissemination to up to 100,000 youth annually. Results of this study inform future intervention studies, interventions aimed at farm youth, and interventions to increase use of hearing conservation strategies, as well as offer a base for developing programs for non-English speaking children.

**Trial registration:**

Clinicaltrials.gov registration CT02472821.

Date of trial registration: 06/09/2015 (retrospectively registered).

## Background

Although farm operators are known to experience among the highest prevalence rates of hearing loss among all categories of workers [1], a less recognized problem of noise exposure and associated negative health effects exists among farm youth. Estimates place the number of children and adolescents exposed to such hazards at 2 million, as farm residents, farm family workers, hired workers, children of migrant or seasonal workers, or farm visitors [2]. Epidemiological evidence demonstrates that farm youth are at disproportionate risk for negative health effects of noise exposure, with risk factors including frequent exposure to high farm noise [3] from an early age [4, 5]. Compounding these risks is exposure to high recreational noise (e.g., firearms, all-terrain vehicles, and personal listening devices). In comparison studies, farm youth have lower hearing ability than their urban peers [6, 7].

While noise elimination is the most preferred method of prevention of negative effects of noise, this approach is often not technically or economically feasible in the farm work environment [8]. Consistent use of hearing protection devices will prevent noise-induced hearing loss and other negative effects of noise [9–11], but their use in the farm work setting is low and often ineffective [12].

There has been some recorded success in improving hearing conservation in the short-term (up to 6 months) among non-farm youth using classroom-based programs [13–15]. As for farm youth, tests of effectiveness of hearing conservation programs for this group have shown uneven success. Marlenga [16] had an exceptionally long follow-up study, reporting that at 16-years post-intervention, participants from the intervention group (*n* = 375) had increased use of hearing protection in agricultural settings and while shooting guns, but there was no difference in use between experimental and control groups in other settings. In a cluster randomized control trial of a classroom training program for 753 rural youth, Berg [17] reported a positive effect on frequency of hearing protection device (HPD) use after 3 years. In a study of 70 high-school-aged FFA members, Khan [18] reported no differences between groups in a test of effectiveness comparing classroom, classroom with smartphone app, and computer-based learning approaches. Conclusions from many of these studies are constrained by methodologic issues. In addition, reach and sustainability of the programs were weak.

Given the challenges of retaining health behavior over time, some authors have recommended use of boosters in education programs [13]. However, previous studies examining the effect of boosters on adult hearing protector use [19] have had mixed results, and there is no published report of their use in addressing noise exposure among youth. The aim of this study was to compare the effectiveness of a community-based interactive youth educational program alone, the program followed by an Internet-based booster, and a no-interaction control.

## Methods

The study consisted of a cluster randomized control trial, consistent with the Consolidated Statement of Reporting Trials [20].

### Participants

Through their Safety Day program, the Progressive Agriculture Foundation provides education and training to over 100,000 children and adults annually through local affiliates, making farm, ranch and rural life safer and healthier for children and their communities [21]. Safety Day is a one-day, hands-on workshop that teaches children safe farm practices. For 1 day each year, many rural schools around the U.S. invite Safety Days volunteers to organize and run fun, interactive, and hands-on learning. These programs give positive attention to safety, provide knowledge about farm safety, encourage parents and children talk about safety, and enhance safety awareness in the community [22–25].

Farm and rural youth, as well as Safety Days instructors and coordinators were sampled. Inclusion criteria for youth participants included enrollment in elementary school grade 4, parental consent, English speaking, and attending a Safety Day event included in the cluster sampling. Inclusion criteria for coordinators included demonstrated ability to successfully coordinate a Safety Day program, interest in the study, opportunity to implement the lesson in the designated study period, and willingness to comply with study procedures. Inclusion criteria for instructors included recommendation by the program coordinator and accepting a hearing health teaching assignment at a Safety Days event included in the cluster sampling.

### Sampling procedures

The study used pre-existing Safety Days protocols for recruitment and consenting youth participants. One or more study team members met with groups of local community program coordinators at required pre-event training sessions (face-to-face or virtually) to introduce the study purpose and methods, and to recruit support for coordinator and instructor involvement.

Recruitment meetings continued until enrollment quotas were met. Coordinators managed recruitment of Safety Days instructors and youth, with support of the study team.

### Measures

The primary outcome measure for this study was self-reported intent to use hearing conservation strategies. Additional outcomes measured included a) knowledge of hearing health, and b) attitudes toward use of hearing conservation strategies. Measures were adapted from Martin et al. [13], for purposes of comparison across studies. There were no changes to plans for eligibility criteria, interventions, data collection, methods of analysis, or outcomes.

#### Intent to use hearing conservation strategies

For comparison purposes, a six-item, multiple-choice instrument successfully used by Martin et al. [13], was used to measure youths’ intent to use hearing conservation strategies. The instrument has a Flesch-Kinkade reading grade level of 5.6, and a reliability index (Cronbach’s alpha) of .64 in the study sample. A sample item from this instrument is, “What would *you* do to protect your ears at a loud concert?”

#### Knowledge of hearing health

The 12-item, multiple-choice instrument to measure youths’ knowledge of noise, its effects on hearing, and hearing conservation strategies was adopted from items used by Martin et al. to measure this concept among elementary students [13]. A sample item from this scale is, “Which sounds can be loud enough to damage your hearing?”

#### Attitudes toward use of hearing conservation strategies

A single multiple-choice item was used to measure attitude influencing use of hearing conservation strategies among youth. The instrument was adopted from a set of items used by Martin et al. [13] The item queries, “Wearing earplugs around your friends (if no one else is wearing them) would be (*very embarrassing* to *not at all embarrassing*).” The instrument has a Flesch-Kinkade reading grade level somewhat higher (11.3) than the other instruments, due to use of the parenthetical phrase “*if no one else is wearing them*” and the multisyllabic word, *embarrassing.* However, in previous use and pretesting, fourth grade students responded to this item without difficulty.

#### Interventions

Intervention 1 consisted of a 20-min face-to-face lesson delivered to small groups (n ~ 8) of fourth graders as part of a circuit of safety lessons. Content included common farm-based and recreational sources of hazardous noise, effects of noise on cochlear hair cells, effect of distance on reducing noise exposure, proper insertion of a child-appropriate insertable hearing protection device, and methods of dealing with peer pressure to increase noise exposure. Methods of instruction included a highly engaging discovery approach, with youth interacting with instructors, peers, and learning materials throughout the lesson, and demonstration and practice of HPD insertion technique.

Instructors recruited for the program were interested community volunteers recruited by the program coordinators, generally with no education or experience in health care. A written lesson plan including learning objectives, key points to emphasize, suggested script, teaching-learning materials, time allotments, and selected teaching materials (noise level meter, pipe cleaners, laminated placards), were provided to each instructor at least 2 weeks prior to the planned implementation. Other supplies (e.g., noisy equipment such as a leaf blower) were locally sourced. A study team member contacted each instructor in advance of the implementation to verify instructor receipt of teaching/learning materials and to offer individualized training and consultation. Fidelity of intervention was monitored by video recording of a sample of sessions; results are reported elsewhere [26].

Intervention 2 consisted of an independent visit to the Dangerous Decibels® online virtual exhibit. The Dangerous Decibels Virtual Exhibit (DDVE) is a free-to-use Web- and game-based set of eight interactive learning activities designed for children to learn about hearing health (www.dangerousdecibels.org/exhibit/virtual-exhibit/). The program is developmentally appropriate, and includes content in sources of sounds, consequences of noise exposure, and prevention of hearing loss. The program is a user-driven activity where youth independently select which activities to pursue, and how long to engage. Highly interactive game-based activities include rating noise sources (e.g., lawn mower) as safe or dangerous, determining the best course of action (i.e., turn it down, walk away, wear hearing protection) in a variety of noisy situations, and choosing a course of action when confronted with social non-support of use of hearing protection. A computer (with Macromedia Flash® and Shockwave®) and Internet connection (e.g., at home, school, or public library) is required for participation. The intervention was delivered to eligible participants following completion of the three-month survey. Participation was monitored by a sign-in system required to access the site.

Interventions 1 and 2 were similar in that they both included common elements of content: common work and recreational noise sources, effects of noise on hearing, and methods of hearing conservation (i.e., turn it down, walk away, use protection). Delivery methods were distinct, with Intervention 1 using a group-based, face-to-face discovery learning approach; Intervention 2 used a set of Web-based interactive educational games.

#### Procedure

Randomization was at the level of community program, with random assignment of programs in an equal allocation ratio to one of three study arms. There were no deviations from the original protocol. The study protocol (#HUM00077214) was approved by the author’s university institutional review board (University of Michigan Institutional Review Board Health Science and Behavioral Science) in advance of data collection, and written parental consent was obtained before student participation.

Assignment of 36 study sites to three study arms was accomplished by simple randomization using R software [27]. After the statistician generated the allocation sequence, the clinical research coordinator (in consultation with representatives of the partner organization) assessed eligibility. The clinical research coordinator enrolled intervention site coordinators (representing clusters), obtained informed consents, and assigned clusters to interventions (lesson only, lesson with booster, or control) based on the randomization sequence. Site coordinators (or their delegates) enrolled youth participants and obtained informed consent from parents. Interventions were administered through trained lesson instructors (Arms A and B) and online (Arm B). The study did not employ blinding procedures, as participants were aware of the program activities in which they were engaged.

### Sample size justification and analysis

The sample size, in particular the number of clusters for this study, was determined by power analysis for a mixed model design using Optimal Design software [28]. The number of clusters was selected to provide 80% power to detect an effect of either intervention with effect size *d* of .48 compared to the effect of the control treatment. This is the effect size on intention to use hearing conservation strategies we found in our preliminary study and is just under what Cohen defined as a medium sized effect. (In pilot testing, the size of the effect on knowledge was even larger.) Other necessary inputs into Optimal Design were ranges of the average number of students per cluster and estimates of the intraclass correlation coefficient (ICC), a measure of the tendency of students in the same cluster to have similar (correlated) responses. To determine the number of clusters needed, we examined average cluster sizes ranging from 20 to 80, and a value of the ICC of .10 (which is a typical value in health education intervention research) [29]. We also adjusted for the possibility of 20% missing posttest data (which we did by reducing the cluster size by that percentage, thus actually testing for cluster sizes from 16 to 64). These last adjustments increased the number of clusters we needed to include. The result of the power analysis was 80% power to detect the target effect size with alpha of .05 two-tailed if we included 36 clusters with an average cluster size of 16. Thus, we designed the study to include 36 clusters divided equally among the three treatments. Target youth enrollment of 576 is based on these calculations and U.S. Agriculture Census data [29, 30].

#### Data analysis

Summary statistics based on mean, standard deviation or frequency were used to characterize the sample distributions at each time point. Since cluster randomized control design was used in this study, linear mixed models were used to test the association between outcome variables (knowledge scores) and intervention effect (fixed effect) where the sites were included as random effects. The PROC MIXED method from SAS software, version 9.4 (SAS Institute Inc., Cary, NC, USA) was used to carry out the analysis. The intervention was claimed to be significant if its *p*-value was less than 0.05 significance level.

## Results

### Participants

Study participants experienced no harms, adverse events, or unintended effects. Study procedures were highly successful in recruitment and retention of study subjects. Primarily, attention to communication with participants, high regard for participant autonomy, careful recordkeeping of participant progress through the study, and fastidious attention to follow-up are credited for these favorable rates.

For each experimental group, the numbers of participants who were randomly assigned, received intended treatment, lost/excluded, and analyzed for the primary outcome are displayed in Table 1.

**Table 1:** Baseline demographic characteristics (*n* = 1979)

	Arm A (baseline/3mo/12mo)	Arm B (baseline/3mo/12mo)	Arm C (baseline/3mo/12mo)
N	662/296/231	680/308/142	637/243/173
Boy/girl	312 / 327	309 / 366	292 / 333
Age	9.77 ± 1.73	9.62 ± 1.65	9.57 ± 2.38
Hispanic/non-Hispanic	119 / 503	112 / 543	97 / 528
Race			
American Indian, Eskimo, Aleutian	17	30	7
White	424	370	497
Asian or Pacific Islander	3	8	4
Black, African American	77	96	19
Other	57	69	57
Not sure	45	85	38

**Fig. 1:**
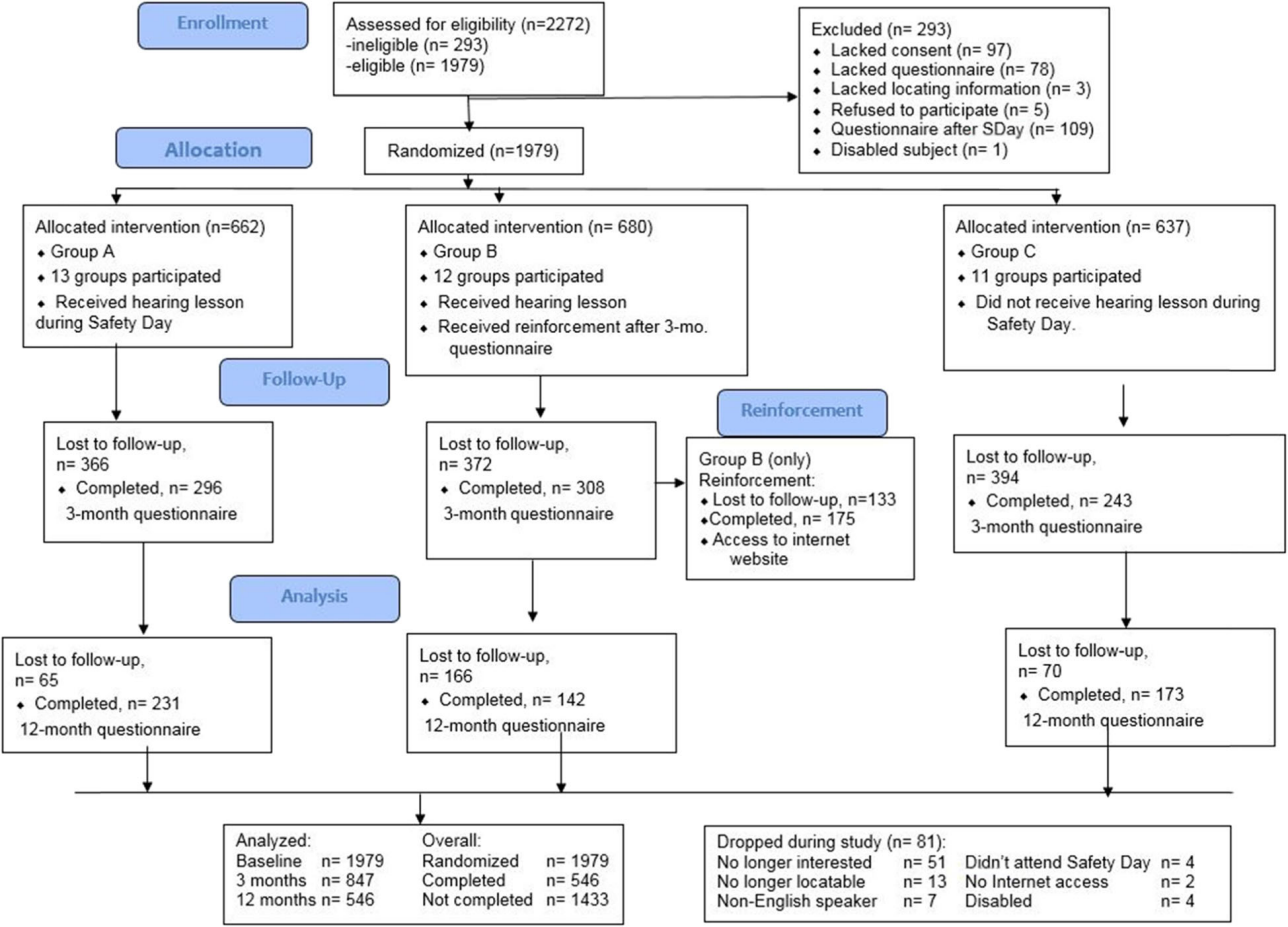
Study Flow Diagram

Recruitment of youth occurred on a rolling schedule beginning in March 2015 and continuing through October of the same year. Participants completed their three-month follow-up questionnaires from June 2015 to June 2016; 12-month data collection occurred between May 2016 and January 2017. Questionnaires were completed by study subjects following reminders by email, voicemail, or postal messages; for those not responding to these contacts, questionnaires were completed by telephone. The 308 subjects randomized to Group B who completed their 3-month questionnaires were invited to access Intervention B via Web; 175 (56.8%) of them accomplished this. A summary of subject progression through the study is included in Fig. 1, Study flow diagram.

### Comparative effectiveness of arms A, B, and C on intent to use, knowledge, and attitude

All comparison groups showed improvement in intent-to-use scores over time. However, comparison of results of intent-to-use measures at 3 months and 12 months using ANCOVA and mixed models, respectively, showed no intervention effect regarding intent to use in either experimental group at either time point.

All comparison groups showed improvement in knowledge scores over time, and knowledge levels were retained over the 12-month study period (Table 2). Comparison of results of knowledge measures at 3 and 12 months using ANCOVA and mixed models analysis showed no intervention effect at 3 months, but a small intervention effect in Arm B (face-to-face lesson with booster) compared to the control group at 12 months. The change in mean knowledge scores appears in the column labeled *Estimate.*

**Table 2:** Knowledge of hearing conservation: Arms B and C (12 months)

Solution for fixed effects						
Effect	Arm	Estimate	Standard error	DF	*t* Value	Pr > |t|
Intercept		0.89	0.02	30.1	51.68	<.0001
Arm	B	−0.044	0.02	57.5	−2.15	0.04*
Arm	C	0	.	.	.	.
Month		0.01	0.00	1548	9.38	<.00

*p < .05

Participants in the experimental Arm B (face-to-face and Internet lesson) showed an increase in favorable attitudes toward hearing conservation approaching significance at 3 months (Table 3). The change in mean attitude scores appears in the column labeled *Estimate.*

**Table 3:** Attitude toward hearing conservation: Arms B and C (3 months)

Solution for fixed effects						
Effect	ARM	Estimate	Standard error	DF	*t* Value	*P*-value
Intercept		2.5838	0.1324	103	19.51	<.0001
Attitude score		0.1650	0.03558	542	4.64	<.0001
ARM	B	0.2244	0.1188	29.7	1.89	0.0688
ARM	C	0	.	.	.	.

Participants in the experimental Arm B also showed a significant increase in favorable attitudes toward hearing conservation at 12 months (Table 4). Again, the change in mean attitude scores appears in the column labeled *Estimate.*

**Table 4:** Attitudes toward hearing conservation: Arm B vs Arm C (12 months)

Solution for fixed effects						
Effect	Arm	Estimate	Standard error	DF	*t* Value	Pr > |t|
Intercept		2.7949	0.065	19.3	48.65	<.0001
Arm	B	0.1584	0.07	22	2.13	0.04*
Arm	C	0	.	.	.	.
Month		0.03173	0.01	1562	6.04	<.00

*p < .05

#### Noise exposure

Participants reported frequent exposure to sources of hazardous noise (e.g., loud sporting events, firecrackers, personal listening devices). For example, nearly 10% (9.3%) of participants reported that they hear loud sound that “made your ears hurt or made you hear ringing sounds.” Most (85%) of participants reported exposure to at least one hazardous noise source one or more times per week. Exposure to multiple sources of hazardous noise were reported, with 85, 63, and 43% of participants reporting exposure to one, two, and three sources of hazardous noise, one or more times per week (Table 5).

**Table 5:** Noise exposure

During the past year, about how often did you do each of the following activities?	Missing	Never	1–3 times/year	1–3 times/month	1–3 times/week	Nearly every day
Gas-powered lawn mower	20	500 (25.5%)	382 (19.5%)	599 (30.6%)	366 (18.7%)	112 (5.7%)
Power tools	28	481 (24.7%)	524 (26.9%)	413 (21.2%)	280 (14.4%)	253 (13.0%)
4-wheeler or motorcycle	22	704 (36.0%)	452 (23.1%)	300 (15.3%)	253 (12.9%)	248 (12.7%)
Fire a gun or be near someone firing a gun hunting or target shooting)	14	867 (44.1%)	589 (30.0%)	273 (13.9%)	137 (7.0%)	99 (5.0%)
Ride in a car with loud music	33	436 (22.4%)	299 (15.4%)	288 (14.8%)	337 (17.3%)	586 (30.1%)
Go to a concert	34	1293 (66.5%)	527 (27.1%)	71 (3.7%)	34 (1.7%)	20 (1.0%)
Use stereo earphones(iPod or MP3 player)	31	276 (14.2%)	178 (9.1%)	286 (14.7%)	492 (25.3%)	716 (36.8%)
Go to a loud sporting event	34	661 (34.0%)	647 (33.3%)	290 (14.9%)	260 (13.4%)	87 (4.5%)
Ride on a tractor	26	1062 (54.4%)	493 (25.2%)	197 (10.1%)	119 (6.1%)	82 (4.2%)
Set off firecrackers	34	572 (29.4%)	1159 (59.6%)	112 (5.8%)	49 (2.5%)	53 (2.7%)
Hear loud sound that made your ears hurt or made you hear ‘ringing’ sound?	27	913 (46.8%)	523 (26.8%)	202 (10.3%)	132 (6.8%)	182 (9.3%)

## Discussion

Results of this randomly-controlled trial illustrated the long-term (12-month) effectiveness of the combined approach of face-to-face and Internet-based educational programs in improving youth knowledge of hearing conservation strategies, as well as their attitudes toward hearing conservation. The success of the face-to-face and Internet-based lessons in improving knowledge and attitudes affirmed the value of this novel, fast-paced, discovery-based learning method with youth. However, the lack of significant change in intent-to-use also affirms results of previous studies illustrating the challenges of making long-term changes in youth hearing conservation-related behavior. It is noteworthy that, although differences between the experimental and control groups in regard to intent to use were not significant, improvements in intent to use were observed in all groups, and changes were retained after 12 months.

Interpretation of these findings are confounded by the potential of a measurement effect. This finding suggests that the baseline and subsequent two waves of testing participants in the control group (i.e., interviewing about hearing conservation strategies, knowledge, and attitudes) may have served as an intervention in itself, influencing participants’ intent to use hearing conservation strategies. Repetition of the study using an alternative study design (e.g., Solomon four-group design) may contribute to untangling this mystery. Moreover, there are limitations to the measurement methods used here (i.e., self-report of intent-to-use), and a corresponding need for improved methods of measuring youth hearing conservation behavior.

The findings of this study are particularly relevant to clinicians, educators, and policy-makers for several reasons. First, the study shows frequent hazardous noise exposure among farm and rural youth. This finding arouses concern regarding not only the effects on hearing, but also the extra-auditory (i.e., somatic) effects of noise exposure, as noise has been related to many highly prevalent health problems. Second, at this time, the effects of noise on the young ear and brain are poorly understood. Consequently, it is prudent for parents, educators, and others to implement the precautionary principle and restrict noise exposure to persons in this age group.

The results of testing of a booster intervention in this study are particularly interesting. The utility of booster interventions in enhancing the effects of hearing conservation educational programs is uncertain, as previous effectiveness studies examining the use of boosters on adult hearing protector use have had ambiguous results. In an effectiveness test of a program designed to improve use of hearing conservation strategies among youth [13], changes in intended use of hearing conservation strategies among fourth grade students participating in the Dangerous Decibels® educational outreach program were not retained after 3 months, suggesting the potential utility of a booster intervention [13]. Based in part on this finding, the study reported here used a booster intervention delivered at 3 months post-intervention. The group receiving the booster showed booster intervention effects in both knowledge and attitudes compared to the comparison groups (i.e., those receiving the face-to-face lesson only and control). However, there was no booster effect in the intent-to-use. This finding confirms results of previous studies demonstrating the recalcitrant nature of behavior change. However, given that the use of booster interventions was successful in changing knowledge and attitudes, this may suggest that these changes may precede behavior change, lending support to offering booster interventions in the long-term interest of hearing conservation.

### Study strengths

The study had a number of strengths. First, reports of noise exposure affirmed the need for noise mitigation programs among farm and rural youth. Large numbers of participants (approaching 10%) reported that they hear loud sounds that “made your ears hurt or made you hear ringing sounds,” and most reported exposure to at least one hazardous noise source one or more times per week.

Many previous tests of interventions have measured effects after short (e.g., 3-month) time periods [13]. However, the study reported here measured effects over a much longer time period (12 months). The robust randomly-controlled trial study design, larger sample size, wide geographic distribution of study sites, and use of appropriate statistical methods also increase confidence in study findings.

This study was one of only a few ever conducted to address accessibility and affordability of services focused on *prevention* of noise-related health problems (e.g., noise-induced hearing oloss). The prevalence of noise-induced hearing loss and other noise-related disorders, their permanence, cost, and related disability speak to the significance of development of effective and sustainable interventions to address this long-standing problem.

Prevention of NIHL among children and adults is included in several national health agendas in the US, including Healthy People 2020 [31, 32], the National Agriculture, Forestry, and Fishing Agenda [33], and the North American Guidelines for Children’s Agricultural Tasks [34]. However, progress in this regard has been slow. Although some small-scale hearing conservation programs for farm and non-farm youth have been successful in changing HPD use behavior over short time periods, the effectiveness of these programs has been limited by lack of systems to deliver programs widely to at-risk youth, and at an effective frequency to maintain change over time [13, 35, 36]. The novel intervention tested in this study was designed to meet the specific needs of the unique population of farm and rural youth while addressing the limitations of previous studies. Farm and rural youth have noise exposures that are unique to them, and have barriers and other attitudes that are unique to this demographic group. Furthermore, the intervention is responsive to the sustainability needs of their rural communities, in that it is low cost and has been deemed sustainable by the communities who used it.

There is a great need to develop, test, and implement programming to protect the hearing of the 2 million farm and rural youth affected by farm noise. In the US, policies designed to protect noise-exposed persons (e.g., OSHA and the Hearing Conservation Standard, youth labor laws) rarely protect farm youth [8, 37–39]. Well-intentioned parents and employers of youth on farms are often ill-equipped to protect their children from these hazards, as they are often not knowledgeable regarding hearing conservation, may not recognize the susceptibility of youth to damage from noise, and are unprepared to supervise youth in when to wear, which types are suitable, where to purchase, and how much to pay for hearing protection devices [38]. For these reasons, children engaged in farm tasks are highly vulnerable to negative effects of noise on their hearing, cognition, and general health and are underserved by programs designed to protect hearing health.

Additional evidence exists to support action to improve programming to protect the hearing health of farm and rural youth. In addition to its effects on hearing, noise exposure, NIHL has a negative impact on the physical and emotional functioning, social life, and employment of the affected person. Aside from its deleterious effects on hearing, noise is also associated with various somatic effects. For example, increases in hypertension and blood cholesterol were attributed to occupational noise exposure [40]. .In addition, NIHL results in heavy social and economic burdens on families and communities from all ethnic and socioeconomic groups. Persons with NIHL frequently experience tinnitus, and are at increased risk for personal injury due to difficulty hearing warning sounds [41]; farm operators with hearing loss are at higher risk for work-related trauma [42]. Noise is also associated with declines in verbal and nonverbal learning and memory [43]. Most affected persons are unaware that they are affected until it is moderately severe [44], reducing the likelihood of early diagnosis and treatment, before significant permanent losses occur. These factors support the need for the development, testing, and implementation of early interventions to protect the hearing health of farm and rural youth.

The study was conducted in geographically dispersed communities, representing a variety of cultural and demographic compositions, suggesting that the program may be successful in a wide variety of English-speaking settings involving fourth graders.

### Limitations

The primary outcome measure of the study, intent to use hearing conservation strategies, was measured using self-report. This measure was selected, in part, because this measure has been used repeatedly in previous intervention studies of youth hearing conservation interventions dating as far back as 1998 [35] and as recently as 2013 [13]. Because study effectiveness measures are based on participant self-report, they are vulnerable to social desirability bias. However, in a nationwide sample of farmers participating in a test of hearing conservation interventions, participants scored low on a standard measure of social desirability reporting [45]. In other reports, self-report and observations were highly correlated [46, 47]. These factors support the use of self-report in measurement of use of hearing conservation strategies. However, the reliability and validity of the intent-to-use measure has not been studied among farm and rural youth.

The hearing health educational program was delivered in the U.S. exclusively in English, and primarily to residential (non-migrant) youth. This is a significant limitation of the study, and an area for future research and program development. Furthermore, because the study enrolled youth primarily in the fourth grade, the appropriateness and effectiveness of the program for younger and older youth is untested.

## Conclusions

In summary, although the problem of noise-induced hearing loss among farm and rural youth is long recognized, and the number of youth affected numbers in the millions, there is a lack of noise mitigation programs with demonstrated effectiveness. This study provides evidence of the effectiveness of a new *evidence-based* program to protect *a high-risk and underserved population of* rural and farm youth from noise-induced hearing loss, where programs are not currently present. Results showed that the combined face-to-face and Internet interventions were more effective in increasing knowledge and improving attitudes toward use of hearing conservation strategies than comparison groups not receiving the dual interventions. Subjective reports found that the program operated well, was enthusiastically received by the host agency, and has been adopted for delivery to up to 100,000 farm and rural youth annually.

This study represents one of very few clinical trials testing the effectiveness of hearing conservation interventions for farm operators or farm youth. Whereas previous trials have been limited in reach and sustainability, these results can be used to inform future intervention studies, interventions aimed at farm youth, and interventions to increase use of hearing conservation strategies. Outcomes of this study provide knowledge necessary to implement and sustain quality and cost-effective services to large numbers of farm and rural youth, a high-risk and underserved population. This study focuses on primary prevention of noise-related conditions, before the onset of noise-induced hearing loss, and before these youth add to the already high public health burden of persons with hearing loss, and thereby maximizes the opportunity for success, as treatments for noise-induced hearing loss are limited and unsatisfactory.

The full trial protocol can be accessed at http://bmcpublichealth.biomedcentral.com/articles/10.1186/s12889-015-2393-y.

## Data Availability

The datasets generated and analyzed during the current study are not publicly available to protect the privacy of the participants, but are available from the corresponding author on reasonable request.

## Abbreviations

FFA: Future Farmers of America
HPD: Hearing protection device
NIHL: Noise-induced hearing loss
NIH: National Institutes of Health
NIDCD: National Institute on Deafness and Other Communication Disorders
NIOSH: National Institute of Occupational Safety and Health
OSHA: Occupational Safety and Health Administration of the U.S. Department of Labor
